# Erianin suppresses hepatocellular carcinoma cells through down-regulation of PI3K/AKT, p38 and ERK MAPK signaling pathways

**DOI:** 10.1042/BSR20193137

**Published:** 2020-07-27

**Authors:** Liang Yang, Yue Hu, Guanbao Zhou, Qi Chen, Zhenshun Song

**Affiliations:** 1 Department of General Surgery, Shanghai Tenth People’s Hospital, Tongji University School of Medicine, Shanghai 200072, China; 2 Department of Hepatobiliary Surgery, Ningbo First Hospital, Zhejiang Province 315010, China

**Keywords:** erianin, hepatocellular carcinoma, p38 and ERK MAPK, PI3K/Akt

## Abstract

**Background:** Hepatocellular carcinoma (HCC) is the dominant pathological type of primary liver cancer and no effective methods are available for its treatment. Erianin is a natural product extracted from *Dendrobium*, which possesses multiple pharmacological activities, including antioxidative and antitumor activity.

**Objective:** To evaluate the anti-HCC activities of erianin and explore its underlying mechanism.

**Methods:** MTT assay and Crystal Violet staining assay were used to select the non-toxic concentrations for the subsequent experiments. The colony formation assay and PCNA fluorescent staining were used to investigate the antiproliferative effects of erianin on human SMMC-7721 and HepG2 cells. Wound healing and transwell test were used to analyze cell migration and invasion. Caspase3 and Tunel staining were used to detect apoptosis. Western blot was used to examine the expression levels of proteins associated with invasion and key proteins in the phosphatidylinositol-3-kinase/protein kinase B (PI3K/Akt), p38 and ERK mitogen-activated protein kinase (MAPK) signaling pathways.

**Results:** Erianin inhibited HCC cell proliferation in a dose-dependent manner. Decreased migration rate and invaded cells were observed with erianin supplement. The expression of invasion-associated proteins in the erianin group was also down-regulated. Besides, more apoptotic cells were observed after erianin treatment. For the molecular mechanism, erianin inhibited the phosphorylation of Akt, ERK and P38 in the PI3K/Akt and ERK/P38 pathway.

**Conclusion:** We demonstrated, for the first time, that erianin inhibited the proliferation, migration, invasion and induced the apoptosis of HCC through PI3K/Akt, p38 and ERK MAPK signaling pathway, indicating that erianin is a promising agent for the HCC treatment.

## Introduction

Hepatocellular carcinoma (HCC), the dominant pathological type of primary liver cancer, is the third-leading cause of cancer deaths worldwide due to its high rate of recurrence and early metastasis [1,2]. According to the latest data from the China National Cancer Registry, the incidence of HCC in China has been the highest over the past years, accounting for >50% of the global incidence of HCC [3]. Despite existing multimodal therapy, including surgical resection, liver transplantation, chemotherapeutic and radiotherapy, the curative effect on patients with HCC is still not as good as anticipated and the prognosis remains poor [4]. Therefore, it is urgent to explore underlying mechanisms of HCC and dig out novel therapeutic schemes to complement or replace the existing modalities.

The occurrence and evolution of HCC is a complex process involving multiple stages, multiple systems and multiple signaling pathways [5]. Selective blocking of tumor cell signaling pathways and disrupting its self-regulating growth regulation mechanism has become a hotspot in the field of modern oncological research. Accumulating evidence suggests that the phosphatidylinositol-3-kinase/protein kinase B (PI3K/Akt) signaling pathway is vital for the growth, metabolism, apoptosis, metastasis, chemotherapy resistance of cancer cells [6,7]. PI3Ks are heterodimers, composed of one p110 catalytic subunit encoded by PIK3CA (p110α), PIK3CB (p110β) or PIK3CD (p110δ) and one p85 regulatory subunit encoded by PIK3R1 (p85α), PIK3R2 (p85β) or PIK3R3 (p85γ) [8]. Akt, a serine-threonine kinase, is directly activated by PI3K to promote cancer cells growth, survival and metabolism. Activated PI3K transfers AKT from the cytoplasm to the cell membrane [9]. Activated p-AKT is incorporated into cytoplasm or nucleus to promote cell proliferation, anti-apoptosis via phosphorylating a series of substrates. P38 and ERK1/2, which are activated by dual phosphorylation, are two major subfamily members of mitogen-activated protein kinase (MAPK) signaling pathway and play essential roles in the regulation of intracellular metabolism, gene expression and integral actions in many aspects, such as cell growth, differentiation, apoptosis [10]. The previous study demonstrated that p38 an ERK1/2 played roles in malignant phenotypes of HCC [11,12]. Therefore, inhibition of the PI3K/Akt, p38 and ERK signaling pathway is an attractive therapeutic strategy for HCC.

Erianin [2-Methoxy-5-(2-(3,4,5-trimethoxyphenyl)-ethyl)-phenol], a natural bibenzyl compound derived from *Dendrobium chrysotoxum* Lindl., has been used as a herbal drug for thousands of years in traditional Chinese medicine (TCM) due to its antipyretic and analgesic effects [13]. Previous phytochemical studies have reported that erianin possessed multiple pharmacological activities, including antioxidative, antitumor activity [14]. In addition, erianin has been reported to inhibit cell proliferation and induce apoptosis in human promyelocytic leukemia HL-60 cells, and human cervical cancer HeLa cells via PI3K/Akt, ERK signaling and mitochondria-based apoptosis pathways [15]. However, whether erianin suppresses the growth of human HCC cells and its underlying molecular mechanisms behind these effects remain unclear. Thus, in the present study, we explored, for the first time, the effects of erianin on HCC cells and its underlying mechanisms, which may bring new hope for HCC treatment.

## Materials and methods

### Cell culture

HCC cells lines (SMMC-7721 and HepG2 cells) were routinely cultured in DMEM (Sigma–Aldrich, St. Louis, MO, U.S.A.) at 37°C, 5% CO_2_ and 95% humidity.

### MTT assay

HCC cells were cultured and seeded on to 96-well plates. After 24 h, cells were treated with erianin (0, 10, 20, 30, 40 and 50 μM) for 24, 48 and 72 h. Then the MTT solution was added to the wells. The absorbance of the sample is read at a wavelength of 595 nm by the microplate reader.

### Crystal Violet assay

The cultured cells (1.0 × 10^5^ cells/ml) were seeded in nine-well plates and incubated overnight in an incubator. Cells were treated with various concentrations of erianin (0, 10, 20, 30, 40 and 50 μM) for 24, 48, 72 h, and then stained with Crystal Violet. The stained cells were thoroughly washed two to three times with tap water. An alignment observation or a monocular photographing was carried out.

### Colony formation assay

The cultured cells were trypsinized, washed with phosphate-buffered saline (PBS), centrifuged, and resuspended in PBS. The cells were seeded into a six-well plate at a density of 1000 cells/well overnight and then treated with DMSO or erianin at different concentrations (10 and 20 μM) for 24 h. After that, cells were washed with PBS and cultured in fresh medium for 10 days. Then, cells were fixed in 4% paraformaldehyde for 60 min at 4°C and stained with Crystal Violet. The colonies that consisted of >50 cells were counted.

### Immunofluorescence staining

SMMC-7721 and HepG2 cells (5 × 10^4^ cells/well) in four-well chamber slides were treated with erianin (10 and 20 μM). Cells were fixed with 4% paraformaldehyde at 37°C for 30 min. Permeabilization of the cells was achieved after incubation with PBS containing 0.1% Triton X-100 for 30 min. The cells were blocked with a buffer containing 5% bovine serum albumin for 1 h. PCNA diluted in 1% BSA was added and incubated overnight at 4°C. After repeated washes with PBS for five times, cells were incubated with a goat-anti-mouse antibody diluted in 1% BSA (1:200) for an additional 1 min. Finally, the cell nuclei were counterstained with Hoechst33258 dyeing solution and incubated at room temperature for 30 min in the dark. Images were obtained using a confocal laser scanning microscope.

### Wound healing assay

The cells were seeded into 12-well plates at a density of 5 × 10^5^ cells per well and then the confluent cells were gently scratched across the whole diameter of the plates with 100-μl pipette tips. Fresh medium was used to rinse the cells for removing the floating cells. Then, the cells were treated with 10 and 20 μM erianin and the other wells were set as control. The images of the wounded area were captured with Leica DMI3000B microscopy.

### Transwell assay

Transwell assays were performed to detect the capabilities of invasion of SMMC-7721 and HegpG2 cells under the treatment of 10 and 20 μM erianin. Matrigel was evenly spread on a transwell chamber filter at 40 μl/well, and the chamber was placed in a 24-well plate. The cell density was adjusted to 5 × 10^5^ cells/ml. A 100 μl cell suspension were seeded in the upper chambers (Corning Costar, Acton, MA, U.S.A.). Next, fresh medium with 10% FBS was added to the lower chamber, and then maintained at 37°C for 24 h. Next, the cells moving to the lower chambers were fixed with 4% paraformaldehyde for 10–15 min, washed with PBS solution, and stained with 0.5% Crystal Violet for 1 min. The stained cells were observed under Leica DMI3000B microscopy and counted with manufactured software.

### Apoptosis detection

The cell density was adjusted to 10^5^ cells/ml and seeded into 48-well plates. The cells were treated with various concentrations of erianin (10 and 20 μM) and cultured for 24 h at 37°C. The cells were collected and then washed with ice-cold PBS for two times, examined by Tunel and caspase3 reagents. The cells were treated with proteinase K for10 min and then the reaction solution was added, 37°C for 30 min, washed with PBS for three times. The apoptotic cells were counted by fluorescence microscopy.

### Western blot assay

The cell density was adjusted to 10^5^ cells/ml and seeded into 48-well plates. The cells were treated with various concentrations of erianin (10 and 20 μM) and cultured for 24 h at 37°C. Total protein was isolated with radioimmunoprecipitation assay buffer mixed with 1% phenylmethanesulfonyl fluoride (PMSF). Then, the cells were harvested and centrifuged at 12000 rpm for 10 min. The supernatants were collected to obtain total proteins, whose concentration was determined using the bicinchoninic acid (BCA) kit. Samples (40 μg) were subsequently subjected to 12% SDS/PAGE gels and transferred to a PVDF membrane. After blocking with 5% non-fat dry milk at room temperature for 2 h, the primary antibodies of anti-E-cadherin (1:1000, 14472, Cell Signaling), anti-N-cadherin (1:1000, 13112T, Cell Signaling), anti-MMP-2 (1:500, BM0569, Boster), anti-MMP-7 (1:1000, ab207299, Abcam), anti-MMP-9 (1:1000, ab38898, Abcam), anti-p-AKT(Thr^308^) (1:500, ab38449, Abcam), anti-p-AKT(Ser^473^) (1:5000, ab81283, Abcam), anti-AKT (1:1000, ab176463, Abcam), anti-PTEN (1:1000, ab26787, Abcam), anti-p-ERK (1:1000, ab201015, Abcam), anti-ERK (1:1000, CS9102, Cell signaling), anti-p38 (1:2000, ab170099, Abcam), anti-p-p38 (1:1000, ab195049, Abcam) were added and incubated overnight. Subsequently, the membrane was then washed in TBS-T and incubated with horseradish peroxidase-conjugated secondary antibodies of goat anti-rabbit IgG HRP (1:2000, ab6721, Abcam). Protein bands were detected by enhanced chemiluminescent reagents.

### Statistical analysis

Statistical analysis was implemented using SPSS18.0 for windows. All data were presented as the mean ± SD, and differences between groups were evaluated by one-way analysis of variance (ANOVA) with Dunnett’s post hoc test. The level of statistical signification was set at *P*<0.05.

## Results

### Erianin inhibits the proliferation of HCC cells

**Figure 1:**
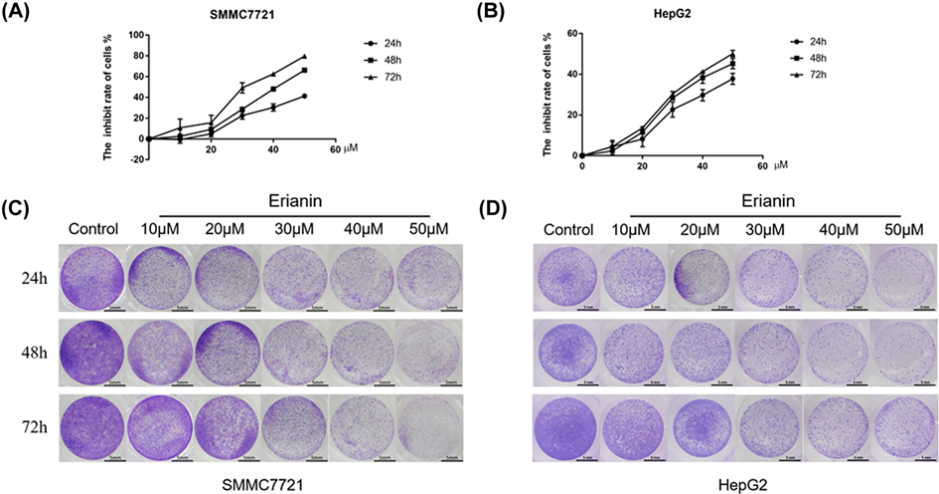
Cytotoxic effect of erianin on the HCC cells (**A**) The MTT assay of SMMC-7721 cells treated with erianin. (**B**) The MTT assay of HegpG2 cells treated with erianin. (**C**) The Crystal Violet staining of SMMC-7721 cells treated with erianin. (**D**) The Crystal Violet staining of HepG2 cells treated with erianin.

To initially observe the cytotoxic effect of erianin on SMMC-7721 and HepG2 cells, MTT assay and Crystal Violet assay were performed. As shown in Figure 1A–D, the proliferation of HCC cells was significantly suppressed when the erianin concentration exceeded 30 μM. Therefore, the concentrations of 10 nd 20 μM were used in the subsequent experiments to preclude the toxic effect of the agent.

**Figure 2:**
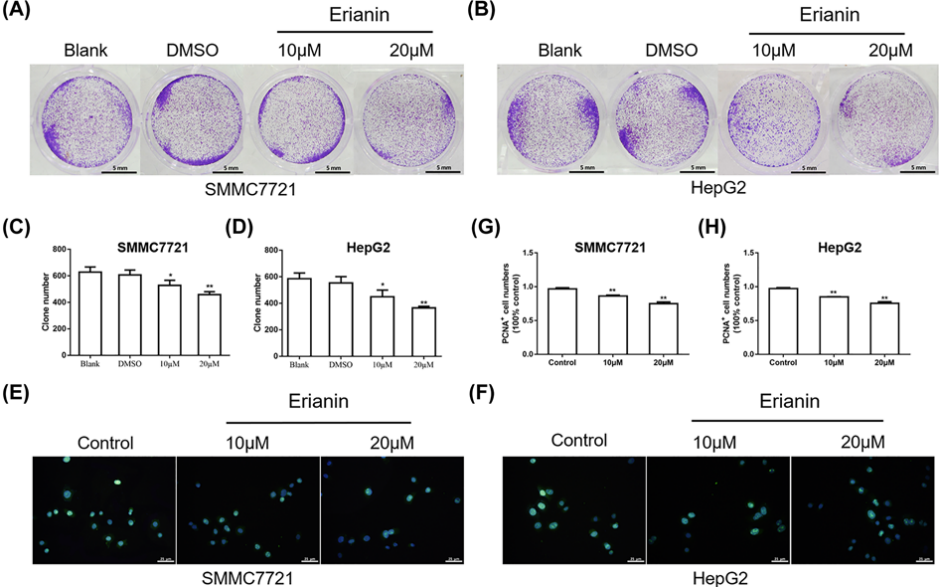
Erianin inhibits the proliferation of HCC cells (**A**) The colony formation assay of SMMC7721 cells treated with erianin. (**B**) The colony formation assay of HepG2 cells treated with erianin. (**C**) The quantitative analysis of clone number in (A). (**D**) The quantitative analysis of clone number in (B). (**E**) Immunofluorescence staining of the PCNA in SMMC7721 cells treated with erianin. (**F**) Immunofluorescence staining of the PCNA in HepG2 cells treated with erianin. (**G**) The quantitative analysis of PCNA+ cells in (E). (**H**) The quantitative analysis of PCNA+ cells in (F). Data are presented as mean ± SD. *P<0.05, **P<0.01 *vs.* DMSO or control group.

In order to further explore the inhibitory effect of erianin on actively proliferating tumor cells, the colony formation assay was performed. As shown in Figure 2A–D, the blank and DMSO group showed a considerable growth of SMMC-7721 and HepG2 cells. The addition of erianin showed reduced clone number in a dose-dependent manner. In addition, PCNA fluorescence staining assay was performed. As shown in Figure 2E–H, erianin inhibited the expression of PCNA in a dose-dependent manner, demonstrating the inhibitory effect of erianin on HCC cell proliferation.

### Erianin inhibits the migration and invasion of HCC cells

To identify the effect of erianin on the migration and invasion of SMMC-7721 and HepG2 cells, wound healing assay and transwell assay were conducted.

**Figure 3:**
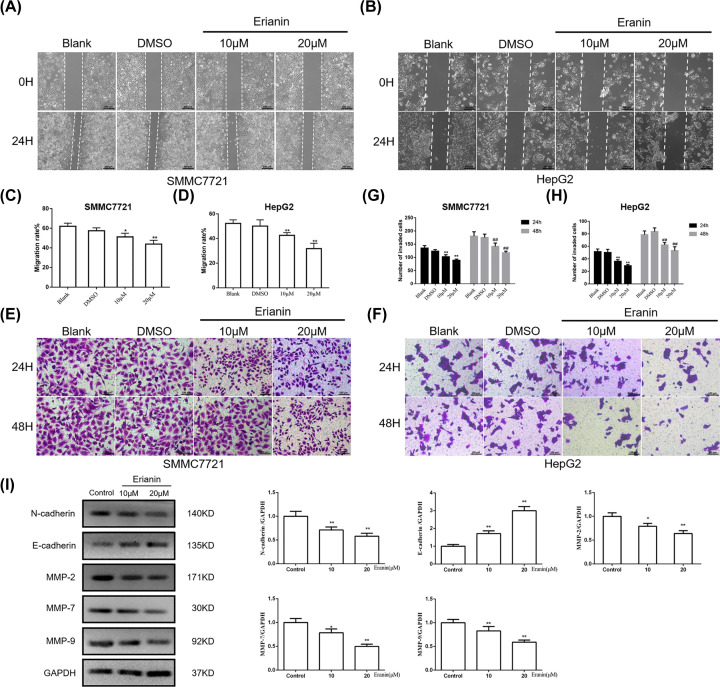
Erianin inhibits migration and invasion of HCC cells (**A**) The wound healing assay of SMMC7721 cells treated with erianin. (**B**) The wound healing assay of HepG2 cells treated with erianin. (**C**) The quantitative analysis of migration rate in (A). (**D**) The quantitative analysis of migration rate in (B). (**E**) The transwell assay of SMMC7721 cells treated with erianin. (**F**) The transwell assay of HepG2 cells treated with erianin. (**G**) The quantitative analysis of the number of invaded cells in (E). (**H**) The quantitative analysis of the number of invaded cells in (F). (**I**) Western blot of invasion-related proteins E-Cadherin, N-Cadherin, MMP2, MMP7 and MMP9 in SMMC7721 cells. Data are presented as mean ± SD. *P<0.05, **P<0.01 *vs.* DMSO or control group.

As demonstrated in Figure 3A–D, after 24-h culture, in contrast with the non-treatment group, erianin markedly delayed the healing processes of HCC cells. The recovery of the wound in the group of erianin at 20 μM was the slowest, indicating erianin significantly inhibited HCC cell migration. In the transwell assay, fewer cells in the erianin treated group invaded through the transwell membrane (Figure 3E–H). Also, the inhibitory effect of erianin on HCC cell invasion showed a substantial dosage-dependent manner.

Since E-cadherin, N-cadherin, MMP-2, MMP-7, MMP-9 are invasion-associated proteins, we further detected the expression of these proteins in the SMMC-7721 cells. As shown in Figure 3I, in contrast with the control group, N-cadherin, an invasion promoter, significantly decreased in erianin groups (*P*<0.01), while the expression of invasion suppressor, E-cadherin, evidently increased (*P*<0.01). Also, the levels of MMP-2, MMP-7, MMP-9 significantly decreased compared with the control group, indicating that erianin suppressed HCC cell invasion.

### Erianin induces the apoptosis of HCC cells

**Figure 4:**
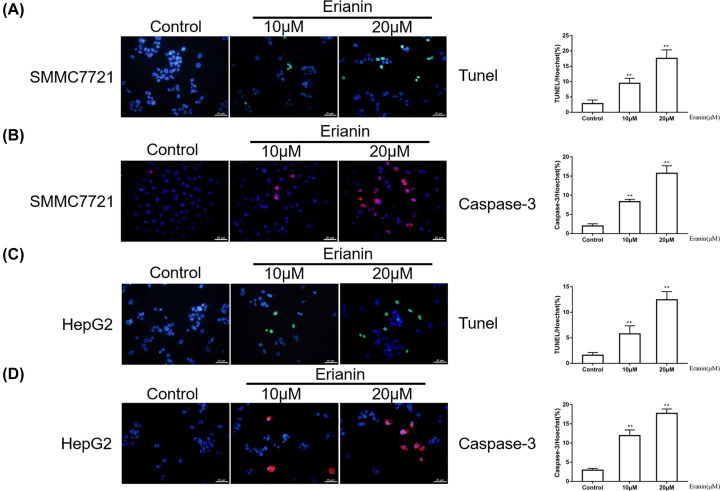
Erianin promotes apoptosis of HCC cells (**A**) Immunofluorescence staining of the Tunel in SMMC7721 cells treated with erianin. (**B**) Immunofluorescence staining of the Caspase-3 in SMMC7721 cells treated with erianin. (**C**) Immunofluorescence staining of the Tunel in HepG2 cells treated with erianin. (**D**) Immunofluorescence staining of the Caspase-3 in HepG2 cells treated with erianin. Data are presented as mean ± SD. **P<0.01 *vs.* control group.

The effect of erianin on apoptosis of SMMC-7721 and HegpG2 cells was detected by Tunel and caspase3 assays. Compared with the control group, the number of apoptotic cells was significantly increased in the erianin treatment group (*P*<0.01) and these changes showed a dose-dependent manner, indicating that erianin promoted the apoptosis of HCC cells (Figure 4A–D).

### Erianin inhibits the PI3K/AKT pathway

**Figure 5:**
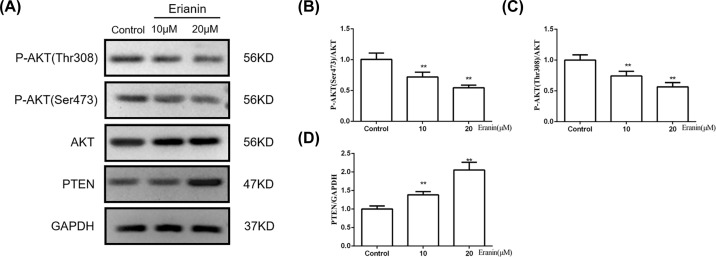
Erianin inhibits the PI3K/AKT pathway (**A**) The expressions of p-AKT(Thr^308^), p-AKT(Ser^473^), AKT and PTEN in SMMC7721 cells treated with erianin. (**B–D**) Quantitative analysis of protein expression in (A). Data are presented as mean ± SD. **P<0.01 *vs.* control group.

To explore the role of the PI3K/AKT pathway in the effect of erianin on HCC cells, we investigated the phosphorylation of AKT after erianin administration by Western blot. As shown in Figure 5A–D, compared with the control group, the expressions of p-AKT(Ser^473^) and p-AKT(Thr^308^) were significantly decreased after erianin treatment. Furthermore, the expression of PTEN, a tumor suppressor gene that inhibited AKT activity, was significantly increased. These results implied that erianin inhibited the activation of PI3K/AKT pathway.

### Erianin inhibits the ERK/p38 pathway

**Figure 6:**
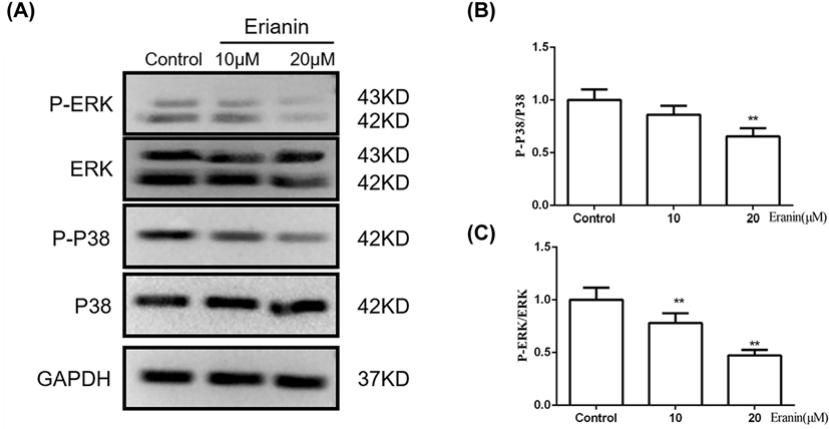
Erianin inhibits the ERK/p38 pathway (**A**) The expressions of ERK, p-ERK, p38, p-p38 in SMMC7721 cells treated with erianin. (**B,C**) Quantitative analysis of protein expression in (A). Data are presented as mean ± SD. **P<0.01 *vs.* control group.

ERK/p38 pathway is essential for the growth and proliferation of tumor cells. Using Western blotting, we investigated the expression of ERK, p-ERK, p38 and p-p38 in different groups. As shown in Figure 6, the expression of p-ERK and p-p38 was significantly decreased after erianin treatment, which indicated that erianin suppressed the activation of ERK/p38 pathway.

## Discussion

The treatment of HCC has been a big challenge in the world. TCM has been considered as a promising source for cancer treatment [16]. Erianin was reported to exert antitumor and antioxidative effects in human cervical cancer HeLa cells [17]. However, the antitumor activity and the potential mechanisms of erianin in HCC have not been assessed. In the present study, we showed, for the first time, that erianin inhibited the proliferation, as well as the migration, invasion and apoptosis of HCC cells via the down-regulation of the PI3K/AKT and p38 and ERK MAPK pathways. Therefore, erianin is a promising agent in the treatment of HCC.

The changes in cell signaling have long been known as the main mechanisms employed by cells in the development and progression of multiple cancers [18]. Significant attention has been focused on the important role of the MAPK pathway since it is critically involved in tumor cell growth metabolism, proliferation, invasion as well as apoptosis. ERK and p38 had been shown to play vital roles in malignant transformation and cancer metastasis and apoptosis via regulating the integrin family of proteins, such as MMPs [19]. In this study, we investigated the effect of MAPK signaling pathway on erianin-mediated anticancer effects. The results showed that erianin inhibited the activation of ERK and p38 via inhibiting their phosphorylation. Therefore, we speculated that the antitumor effect of erianin may, at least in part, result from the inhibition of ERK, p38 MAPK signaling pathway.

Also, several reports showed that the PI3K pathway might be related to tumorigenesis and tumor development [9,20]. According to the clinical research, p-AKT was higher in the tumor (53%) than in cirrhotic tissues (12%) while it was absent from normal liver [21]. PI3K/Akt signaling pathway is also confirmed to be associated with poor survival, high tumor grade, intrahepatic metastasis, as well as vascular invasion in HCC patients. Inhibitors of the PI3K/AKT pathway are under active development as anticancer therapeutics [22]. As expected, we found that erianin decreased the expressions of p-AKT(Ser^473^) and p-AKT(Thr^308^), while increased the expression of PTEN. The results suggested that the antitumor effect of erianin may result from the inhibition of the PI3K/AKT signaling pathway.

## Conclusion

Collectively, erianin inhibits the proliferation, migration, invasion and induces the apoptosis of HCC cells through the PI3K/Akt, p38 and ERK MAPK signaling pathway. Our study provides a rationale for the application of erianin to be a potential agent for HCC treatment.

## Abbreviations

HCC: hepatocellular carcinoma
MAPK: mitogen-activated protein kinase
MMP: matrix metalloproteinase
MTT: 3-(4,5)-dimethylthiahiazo (-z-y1)-3,5-di- phenytetrazoliumromide
PBS: phosphate-buffered saline
PCNA: proliferating cell nuclear antigen
PI3K/Akt: phosphatidylinositol-3-kinase/protein kinase B
TBS-T: tris-buffered saline-tween 20
TCM: traditional Chinese medicine
Tunel: terminal deoxynucleotidyl transferase dUTP nick end labeling
